# A holistic view of innovation incentives and pharmaceutical policy reform

**DOI:** 10.1093/haschl/qxad004

**Published:** 2023-06-20

**Authors:** Rachel Sachs, Loren Adler, Richard Frank

**Affiliations:** School of Law, Washington University in St. Louis, One Brookings Drive, St. Louis, MO 63130, United States; Brookings Institution, 1775 Massachusetts Avenue NW, Suite 824, Washington, DC 20036, United States; Brookings Institution, 1775 Massachusetts Avenue NW, Suite 824, Washington, DC 20036, United States; Brookings Institution, 1775 Massachusetts Avenue NW, Suite 824, Washington, DC 20036, United States

**Keywords:** prescription drugs, innovation, Medicare Part D, Inflation Reduction Act, drug price negotiation

## Abstract

The Inflation Reduction Act is set to transform how Medicare pays for prescription drugs, most notably by enabling Medicare to negotiate for the prices of certain high-cost medications. The pharmaceutical industry argues it will drastically reduce innovation, but a full analysis of its impacts on innovation requires considering not merely the number of new drugs produced but their clinical value. Several features of the negotiation process aim to minimize its impacts on innovation, particularly for drugs with high clinical value. The Biden Administration has also implemented several policies alongside the Inflation Reduction Act, such as the Advanced Research Projects Agency for Health and a National Biotechnology and Biomanufacturing Initiative, that are designed to reward and promote clinically valuable innovation. Taken together, these policies should go a long way toward mitigating any negative impacts on new drug development and may even better promote the development of higher-value drugs.

The August 2022 passage of the Inflation Reduction Act (IRA) is expected to transform the ways in which Medicare pays for prescription drugs, especially by enabling Medicare to negotiate for the prices of certain high-cost medications used by its fifty million beneficiaries, primarily seniors. The pharmaceutical industry by and large opposed the passage of the law, arguing that its drug price negotiation provisions would dramatically harm innovation,^1^ “propel[ling] us light years back into the dark ages of biomedical research,”^2^ pointing to the Congressional Budget Office's (CBO’s) estimate that the law would lead to (modestly) fewer new drugs coming to market over the next thirty years (a 1% reduction).^3^ In our view, however, a full understanding of the IRA's impact on innovation requires considering not merely the number of new drugs that might come to market after the law's passage but the clinical value those new drugs deliver to patients, as well as other policies implemented by the Biden Administration alongside the IRA that are designed to reward and promote clinically valuable innovation. Analyzing these policies in detail demonstrates that IRA opponents’ innovation concerns are overstated and oversimplified, overlooking important dimensions of innovation for patients.

The IRA's negotiation program centers innovation in three main ways, which we categorize as preserving innovation *as a whole*, innovation in *certain classes of products*, and innovation specifically delivering *high value for patients*.

First, the IRA contains provisions aimed at minimizing negative impacts on innovation as a whole. The law directs Medicare drug price negotiations towards innovator small-molecule and biologic drugs that have been on the market for many years without generic or biosimilar competition in an effort to provide companies with time to recoup their investments and earn competitive returns. This commitment is backed by the factors that Medicare must consider during the negotiation process once a drug is selected, including the innovator manufacturer's research and development (R&D) costs for the drug and “the extent to which the manufacturer has recouped research and development costs.”^4^

Putting the negotiation structure in context, for drugs that have been on the market nine to eleven years and twelve to fifteen years, respectively, the IRA requires minimum discounts of 25% and 35% (although larger discounts can be negotiated). Recent CBO analyses estimate that average rebates in Medicare Part D (which primarily governs those medications seniors obtain at a pharmacy, rather than those that are administered by physicians or in a hospital setting) for brand-name drugs are approximately 35%.^5^ Recent research on the financing of cancer drugs also helps illustrate why these IRA provisions serve as protection for innovations that result in high cost prescription drug products. For example, one study found that those products experience a median time to recovery of R&D investments of five years.^6^

Further, the negotiations themselves do not impact a manufacturer's ability to obtain and enforce any intellectual property rights, including patents, trade secrets, and Food and Drug Administration–awarded exclusivity periods, which are typically considered to be core elements of promoting innovation. And even when subject to a negotiated price, manufacturers typically will receive revenues that are higher than if they were facing a generic or biosimilar competitor.

To be sure, in implementing the IRA, Medicare will need to make decisions about how research and development costs will be defined and applied, which will affect this analysis. As one example, it will be important to ensure that Medicare's conception of research and development costs “of the manufacturer” is capacious enough to encompass a range of business models, including manufacturers who choose to in-license compounds from other firms as well as those who develop products in-house. Additionally, important questions also remain about how Medicare should consider the costs of failures. For instance, there are technical issues regarding possible double counting of failures by attribution of failure costs alongside elevated discount rates justified by the elevated risk of R&D failure.

Second, the IRA singles out particular classes of products for special treatment, articulating a legislative judgment that innovation in particular therapeutic areas or by particular entities ought to be protected in different ways. Drugs that have only a single orphan indication are categorically exempt from Medicare negotiations, a policy choice aiming to provide special incentives for innovation in rare diseases. The law also phases in the negotiation program for drugs from small biotechnology companies and creates a temporary price floor for them, giving those companies more time to adjust to the law's implications for their business models.^7^ However, these exemptions are narrowly targeted in an attempt to limit gaming. For instance, although legislators clearly want to protect investments in drugs treating rare diseases, the exemption's limitation to products with only a single orphan indication would prevent manufacturers of drugs for common diseases from avoiding negotiation by obtaining approval for additional orphan indications. This limitation is important, as a recent federal report found that many high-expenditure orphan drugs typically also were approved for more common indications as well,^8^ including several of the top-selling drugs in Medicare.^9^ The net impact on R&D incentives is uncertain.

Third, and most importantly, the IRA aims to limit adverse impacts on innovation that delivers high clinical value for patients, particularly in cases when new products provide only marginal or no clinical benefits when compared with existing treatments. As part of the negotiation process, Medicare must consider a drug's “comparative effectiveness” and “therapeutic alternatives,” as well as whether the drug “address[es] unmet medical needs” for patients.^10^ Drugs that demonstrate a comparative advantage over therapeutic alternatives or that address unmet medical needs—in other words, those that deliver new clinical benefits for patients—ought to command a higher price than those that do not. By limiting negotiated price reductions for drugs that deliver greater clinical benefits, the IRA's negotiation process should maintain strong financial incentives for manufacturers to develop new drugs that represent clinical improvements and to invest in the development of evidence to demonstrate those improvements.

Here as well, regulators do not have perfect information about the relative clinical value of drugs, and key implementation choices will matter to the definition and application of these factors. Who will determine what qualifies as a “therapeutic alternative,” and does it include both drug and non-drug treatment options? In light of companies’ existing weak incentives to produce comparative effectiveness information,^11^ how much of this information is likely to exist in practice? Can the expertise of other administrative agencies, such as the National Institutes of Health (NIH) and its work on comparative effectiveness, be marshaled to support Medicare's work?

More broadly, the ways in which the relevant factors in the negotiation process are weighted and applied against each other will be relevant to these innovation impacts. A negotiation framework that gives greater weight to whether a drug represents a therapeutic advance than to a manufacturer's R&D costs (albeit with recoupment serving as a floor) may create greater relative pressures for manufacturers to engage in high-clinical-value innovation for patients, rather than developing a “me-too” version of an existing product.

In setting and applying these factors through the negotiation process and in defining the precise drugs at issue, the IRA effectively reduces the monopoly pricing that companies can expect to recoup many years after a drug has entered the market, although the IRA does not formally impact companies’ exclusive rights. The application of the negotiation framework itself, therefore, may also realign incentives in a way that increases rewards to companies that engage in the creation of entirely new products relative to the payoffs to engage in activities aimed at extending exclusivity, such as the creation of line extensions with modest clinical value. Indeed, a recent working paper suggests that limiting brand drug firms’ ability to extend market exclusivity for existing drugs may result in *greater* firm-specific research and development investments.^12^

The IRA itself contains provisions outside of the negotiation framework that should impact innovation as well. Several provisions of the law expand insurance coverage of prescription drugs under Part D of Medicare. These include placing a cap on out-of-pocket liability for beneficiaries, reducing the out-of-pocket costs for adult vaccines to zero, and limiting the out-of-pocket costs of insulins to a maximum of $35 per month. Those provisions, in turn, will increase utilization. By increasing utilization, these provisions should increase sales and thereby, in many cases, returns for manufacturers and encourage investment in developing new drugs. On a more modest front, the IRA also doubles the R&D tax credit for small businesses and expands the set of circumstances under which they may use the credit, aiming to enable them to use the credit to the same degree that larger businesses have and increasing their innovation incentives accordingly.^13^ This is especially important given the central role that smaller biotechnology firms are playing in driving innovation.^14^

Additionally, alongside the IRA, the Biden Administration has pursued a range of policies that may impact research and development and should be considered in examining the future of drug innovation. Three such policies—all implemented beginning in 2022—illustrate the range of the administration's interests in innovation policy. First, the administration led the creation of the Advanced Research Projects Agency for Health (ARPA-H), which aims to speed biomedical and health research through building “high-risk, high-reward” capabilities and to drive breakthroughs in diseases such as cancer and Alzheimer's disease.^15^ The administration sought to provide ARPA-H with $6.5 billion in funding over its first three years, as part of a broader effort to increase the budget of the NIH overall (though the omnibus funding package provided only $1.5 billion for three years).^16^ ARPA-H directly complements the innovation-related aspects of the IRA, by increasing investment in disease classes where drug development could lead to significant clinical breakthroughs for patients.

Second, the administration launched a National Biotechnology and Biomanufacturing Initiative in an effort to “coordinate a whole-of-government approach to advance” these fields “towards innovative solutions in health”.^17^ Through this initiative, the administration can engage a wide range of federal agencies to coordinate and support the development of platform technologies and encourage innovation. The Department of Health and Human Services has set out specific steps across its component parts in an effort to support the development and implementation of biotechnology and biomanufacturing technologies.^18^ These initiatives should decrease regulatory barriers to approval, particularly for innovative pharmaceutical products relying on novel manufacturing techniques.

Third and finally, the White House Office of Science and Technology Policy has recommended that federal agencies update their public access policies “to make publications and their supporting data resulting from federally funded research publicly accessible.”^19^ Increasing the amount of taxpayer-funded research that is publicly accessible without costs should have the effect of increasing access to information for researchers—information that can then be used in innovative research and development to benefit patients.

The oversimplified vision of “innovation” and the IRA's relationship to it presented by many of the law's detractors simply do not match the nuanced vision of innovation encompassed either in the IRA or in adjacent policies that the Biden Administration has simultaneously pursued. Although highly uncertain, CBO estimated only a 1% decline in the number of new drugs coming to market over the next thirty years due to the IRA. And features of the negotiation process, in conjunction with the other policies and initiatives discussed in this commentary, should mitigate these impacts on new drug development both broadly and particularly for innovation that matters most to patients. Taken together, it is even possible that these combined changes could increase the development of higher-value drugs, and at the same time reducing costs and increasing patient access.

## Supplementary Material

qxad004_Supplementary_Data
